# Shock wave formation from head-on collision of two subsonic vortex rings

**DOI:** 10.1038/s41598-022-11268-8

**Published:** 2022-05-06

**Authors:** Rachel L. Bauer, Cody J. Thomas, Everett V. P. Baker, Emily M. Johnson, Kelly R. Williams, Martin J. Langenderfer, Catherine E. Johnson

**Affiliations:** Department of Mining and Explosives Engineering, Missouri University of Science and Technology, Rolla, MO USA

**Keywords:** Engineering, Aerospace engineering

## Abstract

Vortex ring collisions have attracted intense interest in both water and air studies (Baird in Proc R Soc Lond Ser Math Phys Sci 409:59–65, 1987, Poudel et al. in Phys Fluids 33:096105, 2021, Lim and Nickels in Nature 357:225, 1992, New et al. in Exp Fluids 57:109, 2016, Suzuki et al. in Geophys Res Lett 34, 2007, Yan et al. in J Fluids Eng 140:054502, 2018, New et al. in J Fluid Mech 899, 2020, Cheng et al. in Phys Fluids 31:067107, 2019, Hernández and Reyes in 29:103604, 2017, Mishra et al. in Phys Rev Fluids, 2021, Zednikova et al. in Chem Eng Technol 42:843–850, 2019, Kwon et al. in Nature 600:64–69, 2021). These toroidal structures spin around a central axis and travel in the original direction of impulse while spinning around the core until inertial forces become predominant causing the vortex flow to spontaneously decay to turbulence (Vortex Rings, https://projects.iq.harvard.edu/smrlab/vortex-rings). Previous studies have shown the collision of subsonic vortex rings resulting in reconnected vortex rings, but the production of a shock wave from the collision has not been demonstrated visibly (Lim and Nickels in Nature 357:225, 1992, Cheng et al. in Phys Fluids 31:067107, 2019). Here we present the formation of a shock wave due to the collision of explosively formed subsonic vortex rings. As the vortex rings travel at Mach 0.66 toward the collision point, they begin to trap high pressure air between them. Upon collision, high pressure air was imploded and released radially away from the axis of the collision, generating a visible shock wave traveling through and away from the colliding vortices at Mach 1.22. Our results demonstrate a pressure gradient with high pressure release creating a shock wave. We anticipate our study to be a starting point for more explosively formed vortex collisions. For example, explosives with different velocities of detonation could be tested to produce vortex rings of varying velocities.

## Introduction

Vortex rings can form not only in air, such as in rocket nozzles and wing tips of airplanes or out the end of a shock tube, but also in water in cavitation bubbles, the wake of a boat and waterspouts^1,2^. Cavitation bubbles can turn into vortices as they travel, and upon impact with the propeller or wall of a ship, break and create shock waves that damage the vessel^14^. As there has been significant research performed on vortex rings in water^3–7,11,15–20^, this paper studies the head on collision of vortex rings in air from an explosively driven shock tube.

Formation of a vortex ring occurs by expelling a slug of fluid through an opening such as the end of shock tube. As the air is moving through the shock tube, it creates a boundary layer on the inner wall of the tube. The shock wave travels out through the center of the tube, creating shear along the boundary layer. This shear causes the air exiting the tube to curl back on itself, pulling in surrounding air, and forming the familiar vortex shape^13^.

Using air as a medium of travel, there have been many studies over the years on vortex ring interaction with a solid boundary. Some studies focus on different angles of vortex collision with a solid wall^4^. Mariani looks at travel distance and the collision of vortex rings with a wall^21^. Others look at boundary layer and wave prorogation around a wall^7,19,21^. A common finding is the production of a wall shock^7,19,21^. As the vortex ring travels towards the wall, a boundary layer is created on the wall opposing the vortex ring. When collision of the vortex ring and the wall boundary occurs, a wall shock is created. This shock travels radially along the surface of the wall^22^.

Using water as a medium for the vortex rings to travel through has provided more success in visualizing and measuring the head-on collision of vortex rings. Studies with water have allowed other researchers to numerically simulate vortex ring collisions as well. Much research focuses on the formation of secondary vortex ringlets after the collision^3,8,9,23^. The Lim and Nickels paper in Nature includes images of the vortex rings reconnecting in a water tank^3^. Once the vortex rings collide, fibers or filaments from each ring break and reconnect with the opposing ring. As the rings are reconnecting, they are forming new vortex rings that travel perpendicular to the original direction of travel of the rings. These two new rings are composed of fibers from both original rings^3,8,9,23^.

Reynolds number is frequently used to characterize flows and in analysis of vortex rings. The Reynolds number is a dimensionless value that shows the ratio of inertial to viscous forces^24^. At low speeds, a flow is smooth and considered laminar. High speeds create turbulent flow with eddies and wakes. Reynolds number is used to characterize these flows. Low Reynolds numbers (under 2000) will produce laminar flows and high Reynolds numbers (above 3500) produce turbulent flows^25,26^. At high Reynolds numbers, the vortex ring collision simply leads to a cloud of turbulence as opposed to reconnected vortex rings^10,27^. Vortex ring collisions at Reynolds numbers below 2000 have been shown to create reconnected rings^3,28–30^. The vortex rings split at the core into two separate vortices then reconnect with the opposing ring^3,28,29^. Although high Reynolds numbers lead to a turbulent cloud, the incident vortex rings have the highest possible kinetic energy^27^. Reynolds number can significantly affect the kinetic energy of a vortex ring. A lower Reynolds number will produce laminar flow and vortex rings with lower kinetic energy. The vortex rings simply travel through the laminar flow and need little energy to continue traveling. At high Reynolds numbers, vortex rings require more kinetic energy to travel. They must pass through turbulence and keep the vortex shape^31,32^.

As Reynolds number increases, small vortices are formed in the trailing area of the incident vortex rings. These small vortices can cause the primary or incident vortex ring to disintegrate more rapidly^31^. Occasionally, small, reconnected vortex rings will appear in the cloud, supporting the previous research of reconnected rings^3^.

Of particular interest are studies of shocks forming in vortex rings. Baird finds that a reverse shock forms in a vortex ring leaving shock tube^1^. This vortex ring has an area of very low pressure and is shown clearly in photographs. The unsteady flow following the shock wave was imaged and revealed these reverse shocks in the vortex ring^1^. Vortex rings have also been shown to have mini shocks inside the rings as they travel through air. As a vortex ring approaches a square plate, a mirror effect occurs. A vortex ring forms on the plate and interacts with the vortex ring traveling toward it. This interaction forms a surface shock wave and small vortex rings on the plate^4^. Minota found that as two vortex rings collide, they produce an inward facing shock, similar to that of a converging–diverging nozzle^33^. As the area of the collision expands, the velocity increases, creating a shock. In a converging–diverging nozzle, as the exit area expands, the flow velocity increases causing a shock^34^. Minota performed this research in air, and used shadowgraphs to capture images of the collision. In his article, he admits that there is no clear view on the images showing the collision of the vortex rings. The simulations provide the majority of information for his article, with no clear experimental images to show the phenomena occurs.

This research uses Schlieren imaging and computer simulations to investigate the head-on collision of two subsonic vortex rings. The experiment was performed at standard temperature and pressure, thus the speed of sound is Mach 1. Schlieren technology was used to visualize the head-on collision of the vortex rings formed from explosively driven shock tube. ANSYS Autodyn was used as a comparison to the schlieren images. The ANSYS simulation depicts colored images as compared to the black and white schlieren images. The images of the collision and resulting shock wave are clearly captured and presented in the following discussion.

## Results and analysis

**Figure 1:**
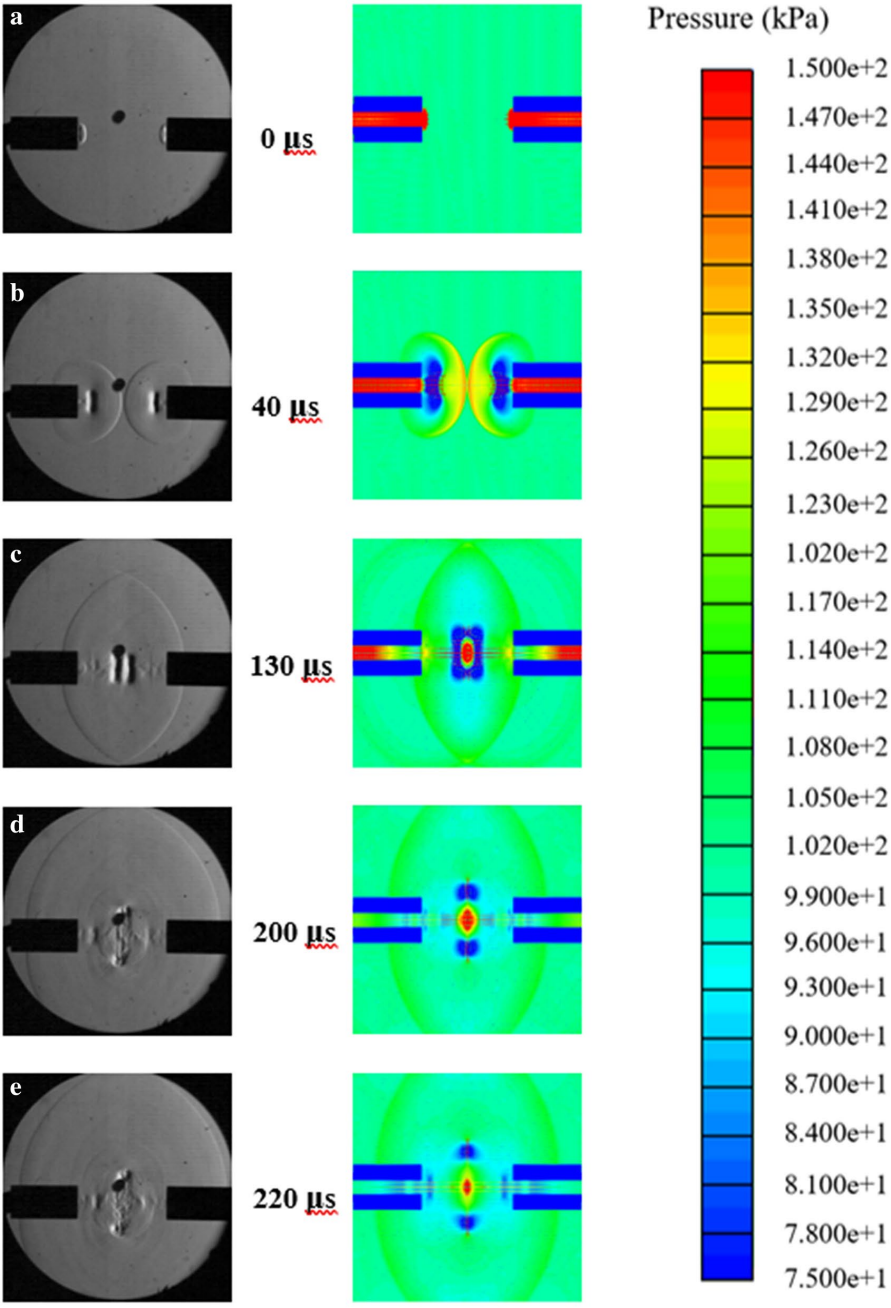
(**a**) The shock waves exiting the tube (**b**) The vortex rings exiting the tube (**c**) The collision of the vortex rings (**d**) The resulting shock wave from the collision of vortex rings e: Turbulent flow moving away from the collision.

Nonelectric lead line was used to create vortex rings from a shock tube. The head-on collision of the vortex rings was captured using schlieren imaging technology and a Phantom camera. Figure 1 shows a timeline of the collision of the vortex rings compared to a simulation between 0 and 230 μs after exiting the tube. Figure 1a shows the instance the incident shock waves leave the tube at 0 μs. Here the vortex ring is just beginning to form. The shock wave leaves the tube at a Mach 1.22 having created shear along the boundary layer inside the tube. The vortex was formed due to the shear causing the air to curl back upon itself behind the shock wave. At 40 μs in Fig. 4b the vortex rings have separated from the tube and the shock waves are about to collide. Here the vortex rings are traveling at Mach 0.66, well below the speed of sound. In Fig. 1c at 130 μs, the vortex rings are shown just before collision. In the simulation image, an area of high pressure can be seen between the vortex rings. This high-pressure air has become trapped between the vortex rings, creating a pressure gradient. After collision in Fig. 1d at 200 μs the high pressure is escaping and creating a shock wave. The schlieren image shows the shock wave beginning to expand radially outward from the collision. The simulation image shows the area of high pressure decreasing as the air is escaping. After the initial collision of vortex rings, and the escaping of high pressure air that creates a shock wave, flow continues to occur from the tubes creating an area of turbulence at the collision site. Figure 1e at 220 μs shows the turbulent flow from the collision. This is the area where previous research has observed reconnection of the vortex rings in water and in simulation. Due to the high Reynolds number in this experimental test, reconnected vortex rings were not observed.

From the point tracking method in the PCC software, change in distance of the wave was found. Using Eq. (1) where x is distance in mm and t is time in seconds, velocity of the wave was calculated. The velocity was converted to Mach then pressure was calculated using Eq. (2) found by McNesby^35^. Kinematic viscosity was calculated assuming ambient air temperature of 22 C^36^. The average speed of the shock wave is Mach 1.12 and the average speed of the vortex ring is Mach 0.42.

1 $$ v = \frac{x}{t}*1000 $$

2 $$ P = \left[ {\frac{{7M_{x}^{2} - 1}}{6}} \right]p_{x} $$

Schlieren video analysis calculated the velocity and pressure of the incident shock waves as well as the vortex produced shock wave. Schlieren has been used successfully in previous studies to provide accurate measurements of vortex rings^37,38^.

**Figure 2:**
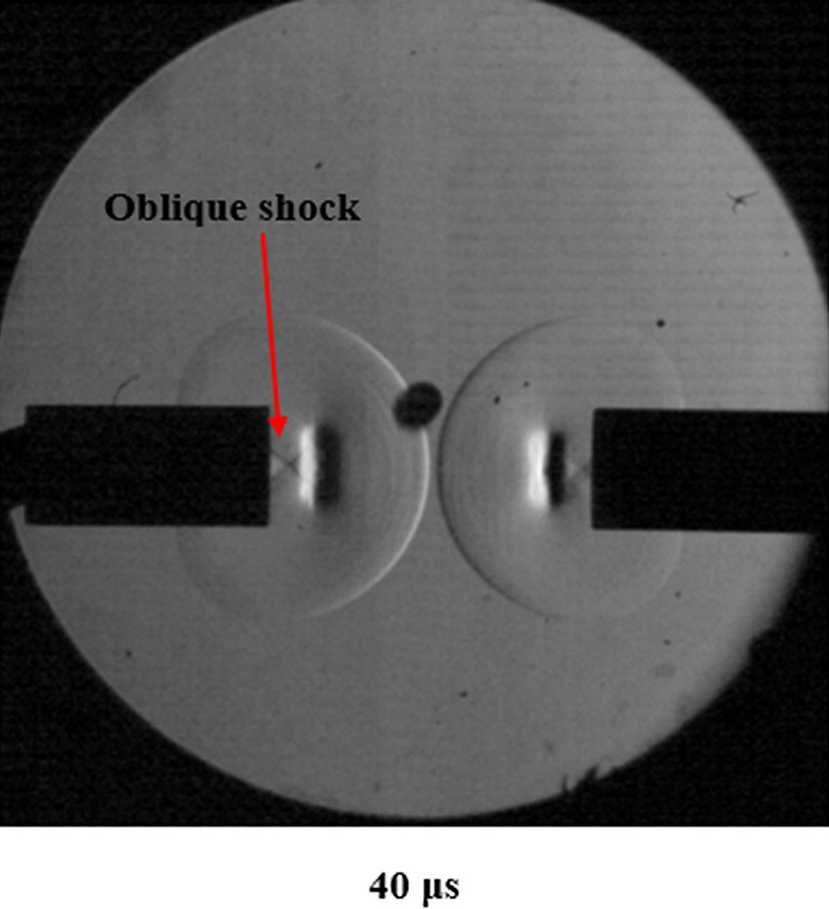
The oblique shock behind the vortex ring created by reflections of shocks in the tube.

Behind the vortex rings in Fig. 2 are lines representative of an oblique shock at Mach 1.6. Figure 2 is an enlarged image of Fig. 4b to show the oblique shock. This shock was calculated using Eq. (3). As the shock dissipates, another normal shock wave exits the tube behind the vortex rings.

3 $$ M_{\infty } = \frac{1}{\sin \propto } $$

$M_{\infty }$ is the Mach of the oblique shock while the $\sin \propto$ is the angle the shock makes as it exits the tube. This shock is seen behind both vortex rings and is comparable to that of a converging–diverging nozzle. The shock in the tube is reflected off the walls of the tube, creating the oblique shock as it leaves the opening.

**Figure 3:**
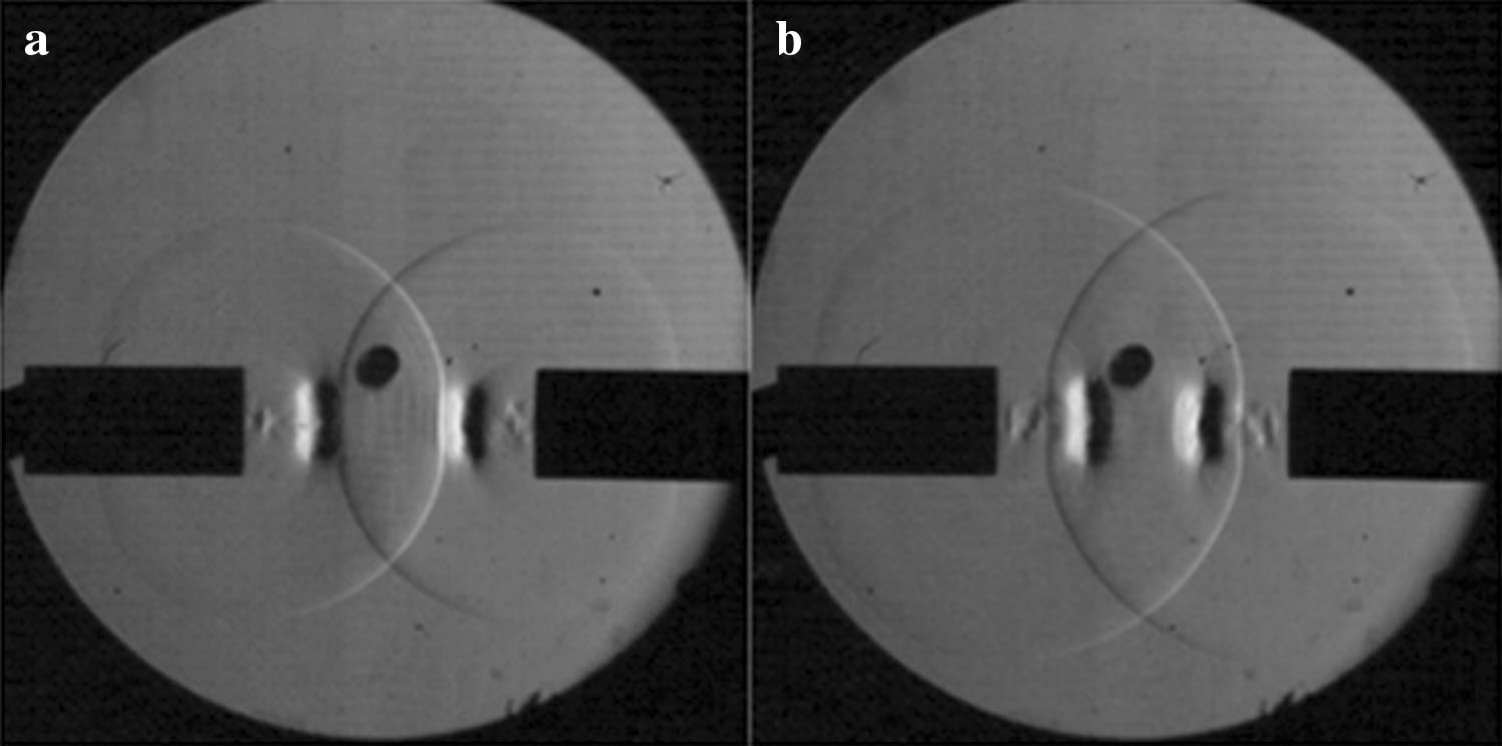
(**a**) Shock wave deformation before vortex collision (**b**) Shock wave leading edge catching up with the rest of the shock wave.

The collision of the incident shock waves at center shows no deformation of the shock waves, however their velocity decreases. As the incident shock waves approach the vortex rings, the shock waves are affected as seen in Fig. 3a. The part of the incident shock wave that impacts the vortex ring is slowed and deformed as it passes through the vortex ring. Only the part of the shock wave that directly impacts the vortex ring is slowed and deformed, the rest of the wave continues traveling outward. As the shock wave exits the vortex ring, it gains velocity and catches up to the part of the shock wave that was not impacted by the vortex ring as seen in Fig. 3b. The rotation of the vortex rings originally slows the shock wave, but then also helps to increase the velocity. The velocity comparisons can be seen in Table 1. These measurements were all taken from the leading edge of the shock wave, which is the part that directly impacts the vortex ring. The other locations of the shock wave that do not impact the vortex ring travel at an average of Mach 1.09. After the leading edge of the shock wave passes through the vortex ring, it can be seen that it does return to the velocity of the rest of the shock wave.

**Table 1:** Shock wave velocity throughout the collision process.

Shock wave leading edge location	Velocity (m/s)	Mach
Initially exiting the tube	419	1.22
After collision with opposing shock wave	349	1.02
At collision with vortex rings	296	0.86
After passing through the vortex ring	371	1.08

**Figure 4:**
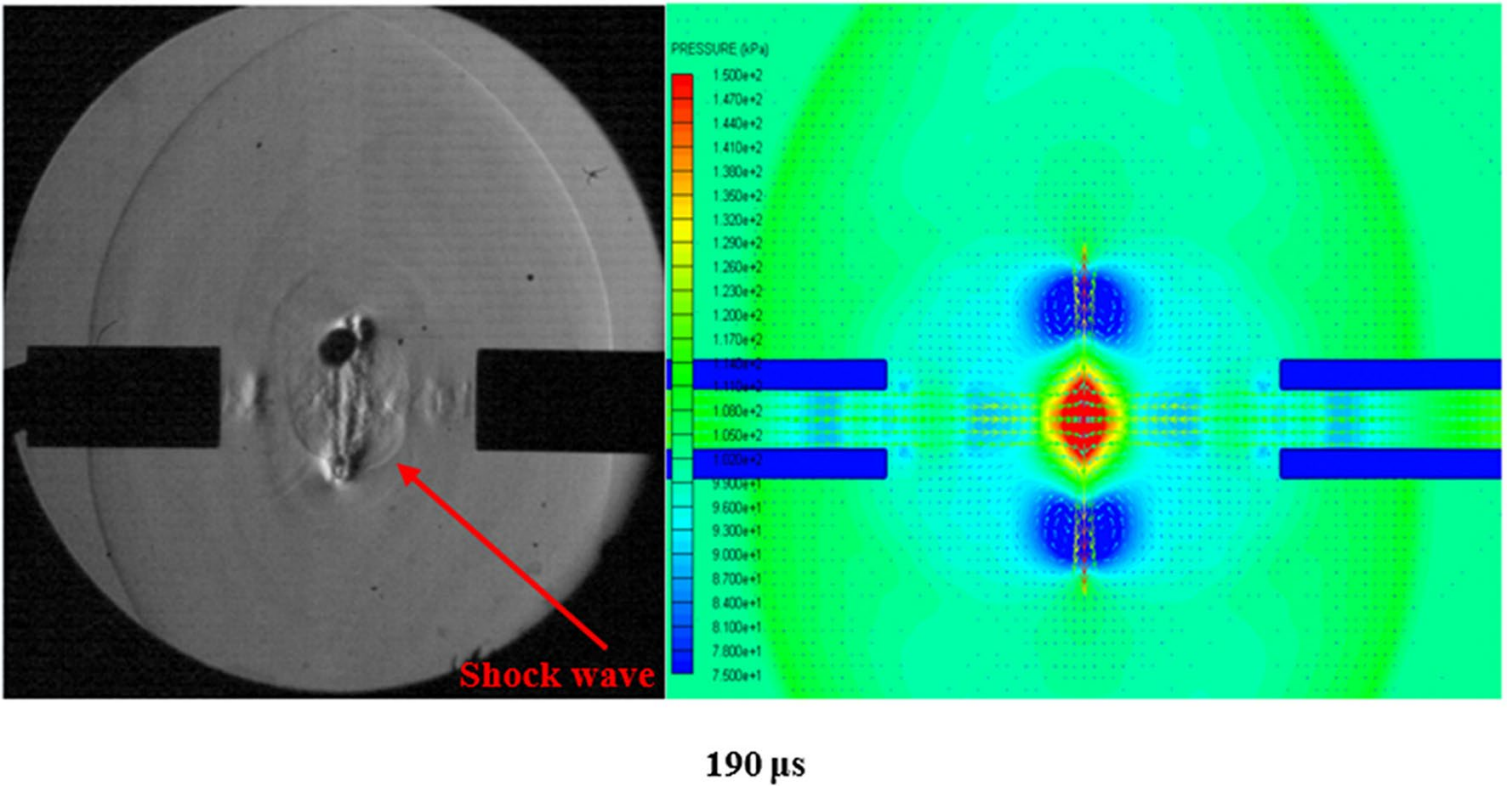
(**a**): Schlieren image of vortex collision with resulting shock wave (**b**) Simulation image of vortex collision.

The vortex rings collide at subsonic velocity but produce a shock wave as a result of the collision. Figure 4a shows the collision and subsequent shock wave expanding in all directions. As the rings move closer together, they trap an area of high pressure between them. Figure 4b shows the simulation of the vortex ring collision, where the red area in the middle is the high pressure air. This high pressure is built up until the collision of the vortex rings. Upon collision, the high-pressure air is released and creates a shock wave due to the velocity of release. In Fig. 6 the shock wave is traveling at Mach 1.12.

**Figure 5:**
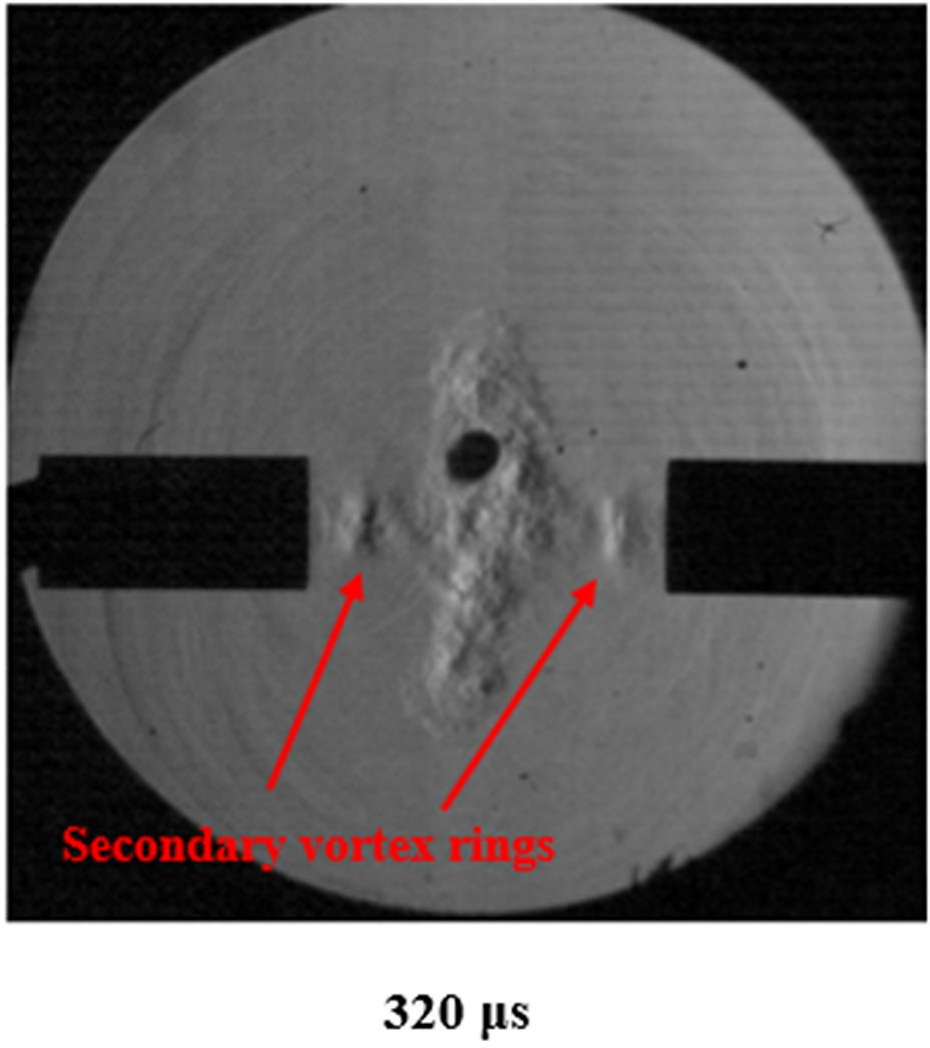
Vortex rings coming from the tube adding to the turbulent cloud.

As the collision of the vortex rings occurs, the initial motion of the rings is stopped. The motion becomes perpendicular to the original direction sending turbulent flow away from the collision. A turbulent cloud perpendicular to the original direction of motion begins to form and move in the same way that reconnected vortex rings have been shown to move in water. Due to the high Reynolds number, a turbulent cloud forms after the collision of vortex rings as opposed to reconnected vortex rings in previous literature^3^. Smaller secondary vortex rings then exit the shock tube. This can be seen in Fig. 5. These smaller rings merge with the initial collision adding to the turbulent field and sending the resulting flow perpendicular to the collision.

The average radial velocity of the incident shock wave and vortex collision shock wave was measured using the PCC software. After converting velocity to Mach, the pressure of the shock waves was calculated using Eq. (2). Table 2 shows the data from the incident shock wave while Table 3 shows the data from the vortex collision shock wave. These measurements were taken as the shock wave expanded radially outward. Both the incident shock wave and the vortex collision shock wave show the shock reach a peak velocity and then begin to slow.

**Table 2:** Incident shock wave data.

Time after incident shock first leaves the tube (μs)	Velocity (m/s)	Mach	Pressure (KPa)
10	384.2	1.12	131.4
30	488.9	1.43	223.3
70	419.1	1.22	159.6
90	419.1	1.22	159.6

**Table 3:** Vortex collision shock wave data.

Time after incident shock first leaves the tube (μs)	Velocity (m/s)	Mach	Pressure (KPa)
220	384.2	1.12	131.4
250	419.1	1.22	159.6
280	349.2	1.02	105.7
310	384.2	1.12	131.4

Equation (4) was used to calculate the Reynolds number of the vortex rings. This equation uses kinematic viscosity of air as opposed to the circulation of the vortex rings and has been used successfully in previous works^3,4^. Calculations were performed using U as the initial translation velocity of the vortex ring, D as the diameter of the vortex ring, and v as the kinematic viscosity of air. The kinematic viscosity of air was calculated assuming ambient conditions of 22 Celsius^36^.

4 $$ Re = \frac{UD}{v} $$

The Reynolds number of the vortices was calculated to be 115,779. Reynolds numbers this high indicate turbulent flow conditions around the vortex rings and after the collision. With the high Reynolds numbers, there will be no reconnection of the vortex rings after collision, just a turbulent cloud. Vortex rings are a major component of turbulent flow, and the high Reynolds number is to be expected in this situation. As the vortex rings move towards each other, they trap an area of high pressure between them. When the vortex rings collide, the high-pressure area is broken and the air rushes out, creating a shock wave.

## Conclusion

The collision of two subsonic vortex rings was shown to result in shock wave generation at the collision point. Experiments using an explosively driven shock tube as well as ANSYS Autodyn simulation were used to visualize the shock waves exiting the shock tubes and the resulting vortex rings. The vortex rings traveled at an average of Mach 0.42 before collision. After collision, the subsonic vortex rings produce a shock wave traveling at Mach 1.12.

The collision of two opposing shock waves produces no visible deformities in the shock waves, but does produce an instantaneous decrease in velocity of Mach 0.05 compared to the velocity directly prior to collision. As a shock wave impacts a vortex ring, the area of the shock wave that hits the ring is slowed and deformed. Upon exiting the ring, the leading edge of the shock wave returns to the same velocity as the rest of the wave.

As the vortex rings rotate quickly, they generate an area of low pressure and temperature around the entire vortex ring. When the two vortex rings move together, they trap an area of high pressure between them. This area of high pressure has nowhere to escape until the vortex rings collide. Vortex rings are two areas of low pressure colliding, while the high pressure air needs to find a way out. This is known as a pressure gradient in atmospheric science and can be used to describe how a vortex ring collision causes a shock wave. The air will move to equilibrium when given the chance, and the collision of the vortex rings provides an avenue for high pressure air to move out of the confinement. Similar to air escaping a balloon when it is popped, the high pressure air will rush away from the vortex rings at a speed high enough to generate a shock wave.

## Methods

A head-on collision of two vortex rings was examined to study the collisions of subsonic structures in air. Schlieren imaging was used to visualize the collision of the vortex rings. The vortex rings were generated using Nonel shock tube and a 3D printed apparatus. The resulting movement and collision of the vortex rings were observed and analyzed, specifically focusing on the supersonic shock generated from the collision of two subsonic vortex rings.

**Figure 6:**
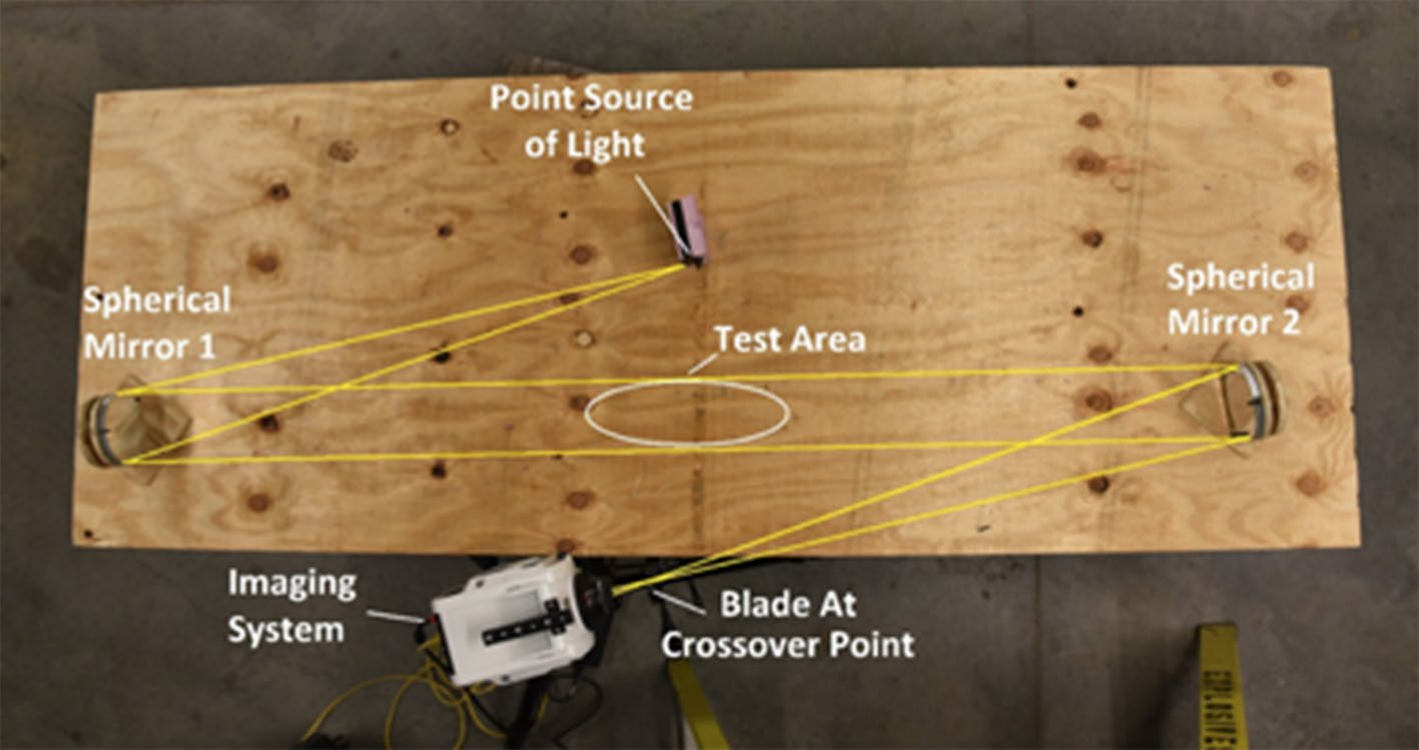
Schlieren test setup.

A two-mirror, Z-pattern Schlieren setup was assembled using a Light emitting diode (LED) as the point light source and a Phantom v2012 high-speed camera with a frame rate of 100,000 fps and resolution of 384 by 384. The mirrors were 135 mm spherical mirrors with a one-meter focal length. The test setup can be seen in Fig. 6.

**Figure 7:**
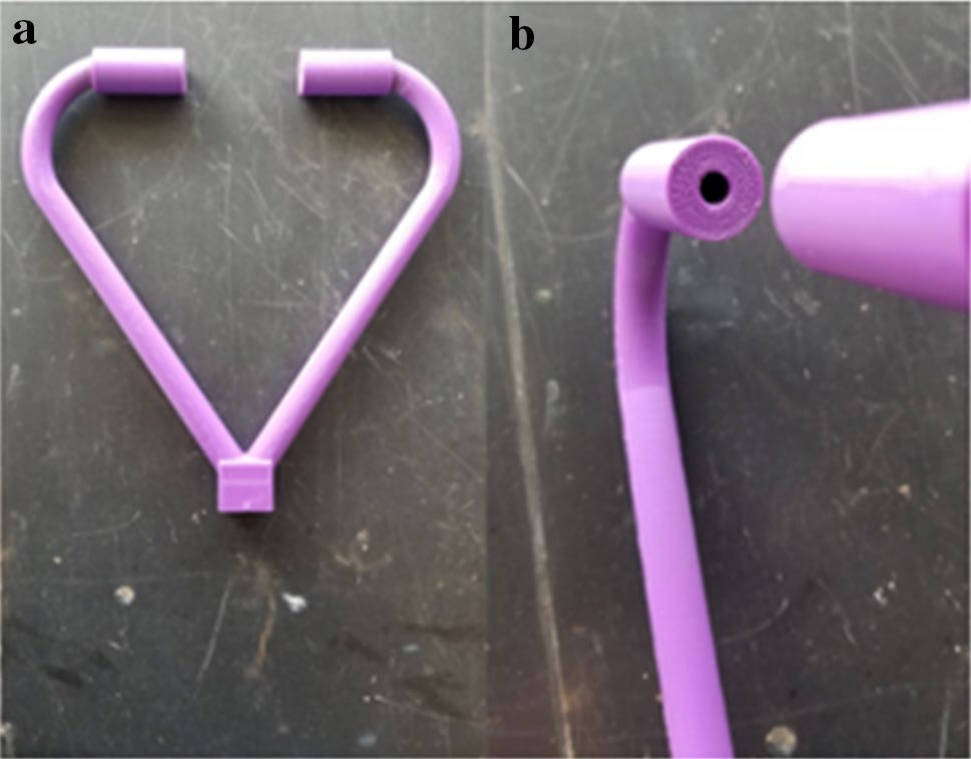
(**a**) The apparatus used to split the shockwave (**b**) the 0.25 inch diameter opening.

The shockwaves emitted were generated via a piece of non electric lead line approximately 1820 mm (6 feet) long and inserted approximately three quarters of the way into a piece of ½" Type L copper pipe that was 812 mm (32 inches) long to act as a circular shock tunnel. The HMX/Al dusted non electric lead line has a velocity of detonation of around 2000 m/s (6500 ft/s)^39^. On the end of the copper tubing was a 3D printed apparatus designed to split the shockwave into two openings that would direct the generated vortex rings onto a collision course. Each end of the shockwave splitting apparatus had a half inch end portion that was straight and allowed fitting of adapters to create the circular vortex rings. The circular exit has a 6.35 mm (0.25 inch) diameter opening. The distance between the openings of the adapters is 50.8 mm (2 inches). The apparatus can be seen in Fig. 7. The Phantom PCC software was used to measure shock and vortex velocity.

**Figure 8:**
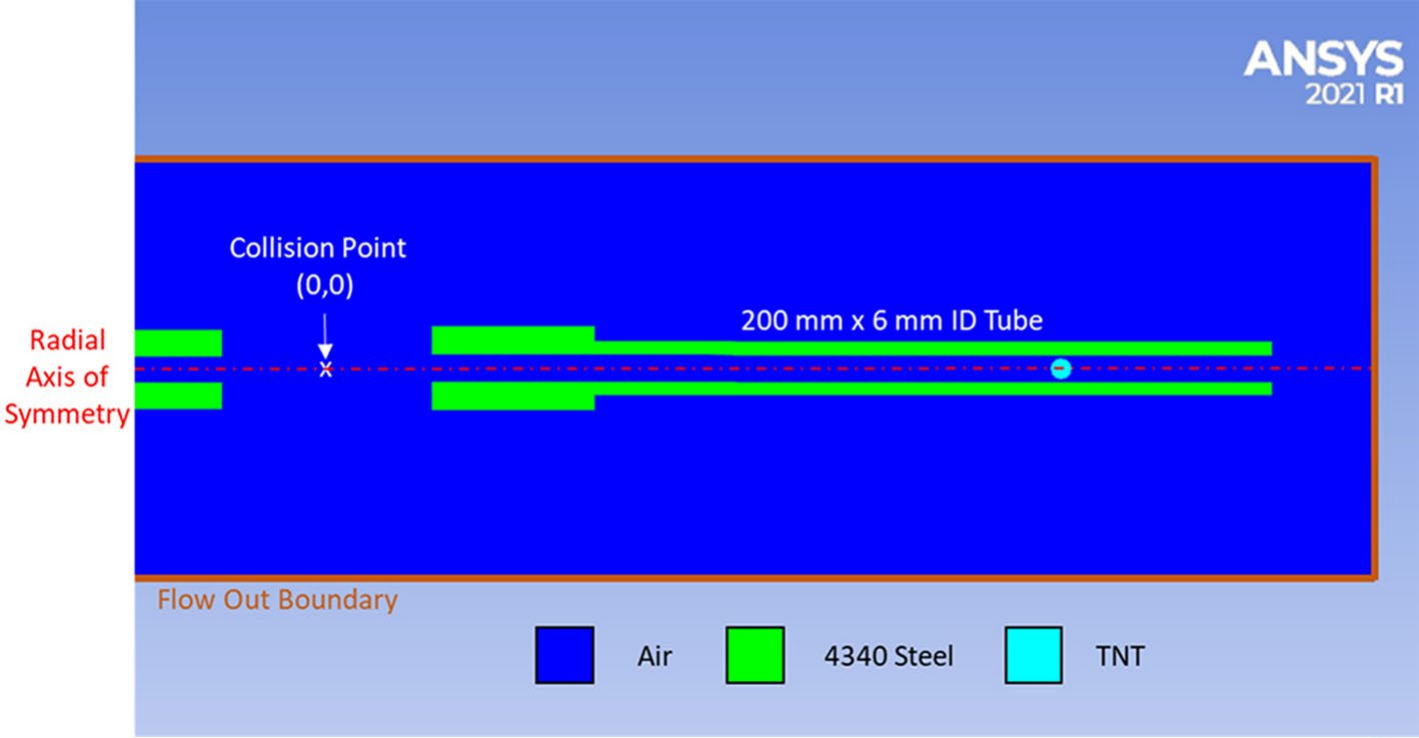
Section view of coupled Lagrangian/Eulerian vortex collision simulation setup mirrored about the axis of symmetry.

A two dimensional axially symmetric coupled Eulerian–Lagrangian simulation was used to model the formation and collision of shock vortices in Ansys Autodyn 2021 R1 and is shown in Fig. 8. Two 4340 steel tubes 6 mm inside diameter × 200 mm long × 6 mm thick were modeled with a 50 × 3 Lagrangian grid along the axis of symmetry using a linear hardening EOS and a Johnson & Cook strength model^40^. The Lagrangian tubes were spaced 50.8 mm apart and were coupled to a Eulerian atmospheric air domain 500 mm long × 50 mm radius with a 2001 × 201 element grid using universal flow out boundaries at the extents of the domain^41^.

To generate a shock wave in the simulation, 1 mg of TNT detonation products were modeled using a JWL equation of state and were isotropically expanded to the 6 mm inside diameter of the tube using a 500 element 1D Eulerian wedge model simulation^42^. The isotropically expanded products were then mapped into the tubes in the 2D axially symmetric air domain and were spaced 50 mm from the end of each tube to produce shock waves in air traveling converging on the center of the Eulerian domain. The simulation was used to visually compare the pressure distribution with the schlieren images. A pressure measurement technique was not used for the simulations as the explosive in the Nonel lead line was not able to be modeled for a direct comparison of the generated shockwave and vortex ring. However, velocity of the incident shock and vortex ring was validated in comparison to the experimental schlieren images.

## Data Availability

The datasets used and/or analyzed during the current study are available from the corresponding author on reasonable request.

## References

[1] Baird JP (1987). Supersonic vortex rings. Proc. R. Soc. Lond. Ser. Math. Phys. Sci..

[2] Poudel S, Chandrala L, Das D, De A (2021). Characteristics of shock tube generated compressible vortex rings at very high shock Mach numbers. Phys. Fluids.

[3] Lim TT, Nickels TB (1992). Instability and reconnection in the head-on collision of two vortex rings. Nature.

[4] New TH, Shi S, Zang B (2016). Some observations on vortex-ring collisions upon inclined surfaces. Exp. Fluids.

[5] Suzuki A, Kumagai I, Nagata Y, Kurita K, Barnouin-Jha OS (2007). Modes of ejecta emplacement at Martian craters from laboratory experiments of an expanding vortex ring interacting with a particle layer. Geophys. Res. Lett..

[6] Yan X, Carriveau R, Ting DSK (2018). Laminar to turbulent buoyant vortex ring regime in terms of Reynolds number, bond number, and Weber number. J. Fluids Eng..

[7] New TH, Long J, Zang B, Shi S (2020). Collision of vortex rings upon V-walls. J. Fluid Mech..

[8] Cheng M, Lou J, Lim TT (2019). Collision and reconnection of viscous elliptic vortex rings. Phys. Fluids.

[9] Hernández RH, Reyes T (2017). Symmetrical collision of multiple vortex rings. Phys. Fluids.

[10] Mishra A, Pumir A, Ostilla-Mónico R (2021). Instability and disintegration of vortex rings during head-on collisions and wall interactions. Phys. Rev. Fluids.

[11] Zednikova M (2019). Experiments on bubble breakup induced by collision with a vortex ring. Chem. Eng. Technol..

[12] Kwon WJ (2021). Sound emission and annihilations in a programmable quantum vortex collider. Nature.

[13] Vortex Rings. https://projects.iq.harvard.edu/smrlab/vortex-rings.

[14] Brujan EA, Keen GS, Vogel A, Blake JR (2002). The final stage of the collapse of a cavitation bubble close to a rigid boundary. Phys. Fluids.

[15] Jha NK, Govardhan RN (2015). Interaction of a vortex ring with a single bubble: Bubble and vorticity dynamics. J. Fluid Mech..

[16] Gharib M, Rambod E, Shariff K (1998). A universal time scale for vortex ring formation. J. Fluid Mech..

[17] Yusupaliev U, Yusupaliev PU, Shuteev SA, Rukhadze KZ (2007). Diffusion anisotropy in a toroidal (ring) vortex in water. Bull. Lebedev Phys. Inst..

[18] Dazin A, Dupont P, Stanislas M (2006). Experimental characterization of the instability of the vortex ring. Part I: Linear phase. Exp. Fluids.

[19] New TH, Zang B (2017). Head-on collisions of vortex rings upon round cylinders. J. Fluid Mech..

[20] Masuda N, Yoshida J, Ito B, Furuya T, Sano O (2012). Collision of a vortex ring on granular material. Part I. Interaction of the vortex ring with the granular layer. Fluid Dyn. Res..

[21] Mariani R, Kontis K, Gongora-Orozco N (2013). Head on collisions of compressible vortex rings on a smooth solid surface: Effects of surface distance variation. Shock Waves.

[22] Minota T, Jiang Z (2005). The Flow-Field Around a Small Square Plate Interacting with the Vortex Flow Released from a Shock Tube. Shock Waves.

[23] Kambe T, Minota T (1983). Acoustic wave radiated by head-on collision of two vortex rings. Proc. R. Soc. Lond. Ser. Math. Phys. Sci..

[24] Reynolds Number. https://www.grc.nasa.gov/www/BGH/reynolds.html.

[25] Barkley D (2016). Theoretical perspective on the route to turbulence in a pipe. J. Fluid Mech..

[26] Rehm B, Consultant D, Haghshenas A, Paknejad AS, Schubert J, Rehm B, Schubert J, Haghshenas A, Paknejad AS, Hughes J (2008). CHAPTER TWO—Situational Problems in MPD. Managed Pressure Drilling.

[27] Lu L, Doering CR (2008). Limits on enstrophy growth for solutions of the three-dimensional Navier-Stokes equations. Indiana Univ. Math. J..

[28] Schatzle, P. R. An Experimental Study of Fusion of Vortex Rings. (California Institute of Technology, 1987). 10.7907/KK00-ZJ41.

[29] Duong VD, Nguyen VD, Nguyen VL (2021). Turbulence cascade model for viscous vortex ring-tube reconnection. Phys. Fluids.

[30] Chatelain P, Kivotides D, Leonard A (2003). Reconnection of colliding vortex rings. Phys. Rev. Lett..

[31] Li Z-Y, Xu Y, Feng L-H, Wang J-J (2019). Synthetic jet vortex rings impinging onto a porous wall: Reynolds number effect. Int. J. Heat Mass Transf..

[32] An X, Jiang L, Hassanipour F (2021). Numerical analysis of air vortex interaction with porous screen. Fluids.

[33] Minota T, Nishida M, Lee MG (1998). Head-on collision of two compressible vortex rings. Fluid Dyn. Res..

[34] Nozzle Design—Converging/Diverging (CD) Nozzle. https://www.grc.nasa.gov/www/k-12/airplane/nozzled.html.

[35] McNesby KL, Biss MM, Benjamin RA, Thompson RA (2014). Optical measurement of peak air shock pressures following explosions. Propellants Explos. Pyrotech..

[36] Air—Dynamic and Kinematic Viscosity. https://www.engineeringtoolbox.com/air-absolute-kinematic-viscosity-d_601.html.

[37] Mizukaki T (2010). Visualization of compressible vortex rings using the background-oriented schlieren method. Shock Waves.

[38] Kainuma M, Havermann M, Sun M, Takayama K, Jiang Z (2005). Effects of the Shock Tube Open-End Shape on Vortex Loops Released from It. Shock Waves.

[39] Nonel Lead Line.

[40] Johnson GR, Cook WH (1985). Fracture characteristics of three metals subjected to various strains, strain rates, temperatures and pressures. Eng. Fract. Mech..

[41] Rogers GFC, Mayhew YR (1995). Thermodynamic and Transport Properties of Fluids: SI Units.

[42] Lee, E., Finger, M. & Collins, W. *JWL equation of state coefficients for high explosives*. UCID--16189, 4479737. 10.2172/4479737. http://www.osti.gov/servlets/purl/4479737/ (1973).

